# Selecting Milk Spectra to Develop Equations to Predict Milk Technological Traits

**DOI:** 10.3390/foods10123084

**Published:** 2021-12-11

**Authors:** Maria Frizzarin, Isobel Claire Gormley, Alessandro Casa, Sinéad McParland

**Affiliations:** 1 Teagasc, Animal & Grassland Research and Innovation Centre, Moorepark, P61 P302 Fermoy, Ireland; maria.frizzarin@ucdconnect.ie; 2 School of Mathematics and Statistics, University College Dublin, D04 V1W8 Dublin, Ireland; claire.gormley@ucd.ie (I.C.G.); alessandro.casa@ucd.ie (A.C.)

**Keywords:** midinfrared spectroscopy, neighbours, local changepoint analysis

## Abstract

Including all available data when developing equations to relate midinfrared spectra to a phenotype may be suboptimal for poorly represented spectra. Here, an alternative local changepoint approach was developed to predict six milk technological traits from midinfrared spectra. Neighbours were objectively identified for each predictand as those most similar to the predictand using the Mahalanobis distances between the spectral principal components, and subsequently used in partial least square regression (PLSR) analyses. The performance of the local changepoint approach was compared to that of PLSR using all spectra (global PLSR) and another LOCAL approach, whereby a fixed number of neighbours was used in the prediction according to the correlation between the predictand and the available spectra. Global PLSR had the lowest RMSEV for five traits. The local changepoint approach had the lowest RMSEV for one trait; however, it outperformed the LOCAL approach for four traits. When the 5% of the spectra with the greatest Mahalanobis distance from the centre of the global principal component space were analysed, the local changepoint approach outperformed the global PLSR and the LOCAL approach in two and five traits, respectively. The objective selection of neighbours improved the prediction performance compared to utilising a fixed number of neighbours; however, it generally did not outperform the global PLSR.

## 1. Introduction

Fourier transform midinfrared spectroscopy (MIRS) is a non-disruptive technique, routinely used in the analysis of both bulk tank and individual animal milk samples to quantify the fat, protein, lactose, and casein concentration [1]. Partial least squares regression (PLSR) analysis [2,3] is the principal statistical method used to relate MIRS spectral data to a trait, as PLSR can handle collinear and high-dimensional datasets. Partial least squares regression is efficient in phenotype prediction when spectral data originate from the same source material, for example milk, and when the relationship between the spectral information and the reference data is linear [4]. Recently, the use of PLSR to relate milk spectral data to various milk and animal traits has been challenged by alternative machine-learning methods [5,6,7]. When non-linearity between the spectra and the phenotype is present, methods such as artificial neural networks (ANN) [8,9], support vector machines [10], and local approaches [11] have been applied.

Local approaches, as reviewed by Perez-Marin et al. [12], aim to identify the most similar spectra to the target spectrum (i.e., the predictand) and then use only these similar spectra (termed neighbours) for phenotype prediction. The principle of the local approach assumes that some spectra in the dataset might be more informative than others and that the utilization of the less informative spectra during the PLSR analysis may cause a reduction in the prediction performance. Therefore, identifying and using the most similar spectra to a predictand can potentially improve the accuracy of the PLSR prediction [13,14,15]. In particular, the local approach has been shown to be a powerful method in heterogeneous datasets in which subgroups of spectra are present. Using a dataset comprising bakery products, mixed feed, poultry feed, and soya products, Berzaghi et al. [13] showed a reduction in the standard error of prediction using the local approach for all the constituents in the dataset.

Different methods have been developed to select neighbours, as well as to relate the spectra to the phenotype [12]. Naes et al. [16] developed an approach called locally weighed regression. The approach identified a fixed number of neighbours based on the Mahalanobis distance applied to the principal components (PCs). Then, it used a simple PC regression, applying different weights to the PCs according to their correlation with the phenotype value, as well as to the neighbours based on their distance from the predictand. Conversely, Shenk et al. developed the LOCAL approach [17], whereby the set of neighbours was identified based on the correlation between the predictand and the available spectra and used in PLSR as predictors of the phenotype associated with the predictand. In the studies of both Naes et al. [16] and Shenk et al. [17], different numbers of neighbours were tested and the best number of neighbours was identified and applied to all the observations in the dataset. While demonstrably better than a global approach, the performance of such local approaches could potentially be improved by tailoring the number of similar neighbours used to the predictand in question. In particular, improving the prediction of traits in outlying spectra is of interest.

Similar to the local methods, cluster methods also use subgroups of samples in the dataset to develop multiple prediction equations [18,19,20]. However, in the local approach, different neighbours are identified for each predictand, whereas cluster methods identify a fixed number of groups (or clusters) in the dataset, where each cluster is characterised by similar samples. Then, a single prediction equation is developed for each cluster, using all the samples belonging to that cluster. Different methods for cluster identification can be used, as outlined by Franzoi et al. [18], who identified clusters according to the trait values or according to the spectra characteristics. 

In this study, we developed a novel local approach, called the local changepoint approach, and applied it to a data set of milk spectral data to predict a suite of milk technological traits. The performance of the local changepoint approach was compared to that of the LOCAL approach developed by Shenk et al. [17], and to that of the global approach which uses all available spectra. The local changepoint approach followed that proposed by Naes et al. [16] to quantify spectrum similarity; however, the number of neighbours was selected in an objective, data-driven manner, using changepoint methodology [21]. The similarity between the predictand and all spectra was calculated using the Mahalanobis distance on the spectra PCs. The changepoint approach, which is able to identify changes in the mean of a vector (in this case the vector of the Mahalanobis distances), was then used to identify the best number of neighbours. Partial least squares regression was applied only to the selected neighbours of the predictand. The aim of this study was to compare the prediction performance of the novel local changepoint approach to the LOCAL and global PLSR approaches in the context of milk spectra and technological traits, with particular interest in the improved prediction accuracy of spectra at the edge of the PC space.

## 2. Materials and Methods

### 2.1. Data 

The dataset used in the present study has previously been described in detail by Visentin et al. [22]. Between August 2013 and August 2014, a total of 713 individual milk samples from 622 cows were collected from 7 different Irish research herds. The samples were collected during morning and evening milking from Holstein–Friesian, Jersey, and Norwegian Red cows, as well as their crosses. The samples originated from animals in different stages of lactation across different parities, and the animals were fed a predominantly grass-based diet with occasional concentrate and grass silage supplementation. The sample analyses were all conducted using the MilkoScan FT6000 (Foss Electronic A/S, Hillerød, Denmark), producing 1060 transmittance data points in the midinfrared light region. The traits considered in the present study included the milk technological traits of rennet coagulation time (RCT), curd-firming time (k20), curd firmness at 30 and 60 min (a30, a60), casein micelle size (CMS), and pH. A Formagraph (Foss Electronic A/S, Hillerød, Denmark) was used to quantify the milk coagulation properties and a SevenCompact pH meter S220 (Mettler Toledo AG, Greifensee, Switzerland) assessed milk pH. A Zetasizer Nano system (Malvern Instruments Inc., Worcester, UK) was used to determine the casein micelle hydrodynamic diameter.

### 2.2. Data Editing

All analyses were conducted using the statistical software R 3.6.1 [23]. To satisfy the assumption of independent observations under PLSR, where multiple records existed for an animal, only one observation was retained. Specifically, for each animal, the observation with the greatest Mahalanobis distance between the principal component scores (PCs) of the observation and the average PCs scores of the entire dataset was retained in order to maximise variability in the dataset. From each spectrum, the high-noise-level regions corresponding to the spectral regions between 1580 and 1710 cm^−1^, between 2990 and 3690 cm^−1^, and >3822 cm^−1^ were removed, leaving a total of 531 wavelengths for analysis. The wavelengths were transformed from transmittance to absorbance by taking the log_10_ of the reciprocal of the transmittance. No further pre-processing was applied to the spectral data. The outliers for each trait were identified as those observations >3 standard deviations from the mean of the respective trait; such observations were subsequently removed from the analysis. Only spectra with information for all the traits of interest were retained. The final dataset comprised 348 samples, whose characteristics are summarised in Table 1. 

### 2.3. Local Methods

#### 2.3.1. The Local Approach

The local approach was developed by Shenk at al. [17]. This methodology consists of identifying the number of neighbours according to the Pearson correlation between the predictand and the other spectra in the dataset. After neighbour identification, a PLSR equation is run to predict the phenotype of the predictand. A range of numbers of neighbours and PLSR factors are considered. Here, subsets of 25, 50, and 100 neighbours were tested. For each group of neighbours, 5, 10, and 20 PLSR factors were tested.

#### 2.3.2. The Local Changepoint Approach

Similar to the Naes et al. [16] approach, the similarities between spectra were calculated using the Mahalanobis distance applied to the PCs of the spectra. The first 4 PCs were used, as they explained 98.2% of the variability in the dataset when the spectra were standardised (centred and scaled) before calculation of the PCs, and a variability greater than 99.9% when spectra were not standardised before calculation of the PCs. To assess the influence of standardising the spectra on the selection of the neighbours and on the subsequent trait prediction, the following analyses were conducted twice, both using the PCs after standardising the spectra and using the PCs without standardising the spectra. Increasing the number of PCs above 4 did not result in changes to the set of neighbours selected. 

To determine the optimal number of neighbouring spectra to use for each predictand, a changepoint analysis was employed [24,25]. Based on a vector of values, a changepoint analysis aims to detect the point or points at which the vector splits into two or more segments with different statistical properties (e.g., different means). For example, when a single changepoint is to be identified, the problem is reformulated as a hypothesis test where the model corresponding to no changepoint is compared to the model with a single changepoint through a likelihood-ratio-based testing approach. On the other hand, when searching for multiple changepoints, the identification is based on the maximisation of a penalised log likelihood function. The penalty is considered to avoid overfitting, possibly leading to the detection of too many changepoints. A changepoint analysis provides not only indications about the presence of changepoints, but also estimates their location in the vector of values.

In the present study, the vector of values was the Mahalanobis distances, arranged in increasing order, between the predictand and the other spectra in the dataset. This approach facilitated a data-driven, automatic, and individualised procedure to select the number of neighbours for each predictand. Different methods for changepoint identification were tested, such as the at most one change (AMOC [24]) and the pruned exact linear time (PELT [21]) methods. The AMOC method identifies only a single changepoint in the provided vector, while the PELT method identifies multiple changepoints. After preliminary analyses, the PELT method was selected as more suitable for the analyses performed in the present study, as it was less sensitive to possible noise in the dataset, and the AMOC approach selected too many neighbours. The changepoint analyses were performed using the changepoint package in R [25]. The PELT method can use different penalties for identifying the changepoints and, in the present study, the Schwarz information criterion (SIC [26]) and modified Bayesian information criterion (MBIC [27]) penalties were tested. Both penalties share the same rationale, as they impose stronger penalisations to models with a larger number of parameters (i.e., changepoints). The MBIC penalty can be seen as a refinement of the SIC, specifically introduced to overcome some of its issues in a changepoint detection framework. To ensure sufficient data were available during the successive PLSR analyses, in particular to identify the best number of PLSR factors, 20 neighbours were defined as the minimum number of neighbours to be used in PLSR; thus, the first changepoint that selected at least 20 neighbours was used.

### 2.4. Prediction Equations

A global PLSR prediction equation was developed for each trait separately using the entire edited data set with the R package pls [28]. A total of 348 global PLSR equations were developed (1 equation for each observation in the dataset). For each predictand, all the remaining samples in the dataset were used to develop the PLSR equation, and leave-one-out (LOO) cross validation (CV) was used to determine the number of PLSR factors. These equations were considered a benchmark against which to consider the performance of the two local approaches. 

For both the LOCAL and local changepoint approaches, only selected neighbours were used for prediction. Separate equations were developed, wherein different sets of neighbours were used to develop the equation according to the different settings employed: for the LOCAL approach, according to the different numbers of neighbours and PLSR factors tested, and for the local changepoint approach, after standardising the spectra or not, or according to the penalty employed. The LOCAL and local changepoint PLSR equations differed in the way the PLSR factors were identified. During the LOCAL analyses, a fixed number of PLSR factors were employed for each group of neighbours, while during the local changepoint PLSR analyses, the number of PLSR factors was identified for each group of neighbours using LOO CV. In total, 9 LOCAL equations and 4 local changepoint equations were developed for each of the 6 traits investigated. 

### 2.5. Measuring Prediction Performance

The prediction performance of the 14 developed equations was assessed using the root mean square error of the validation samples (RMSEV), the correlation between the true and the predicted trait values (r), and examination of the residuals.

## 3. Results

### 3.1. Prediction Performance

Table 2 reports the RMSEV and the correlation between the true and the predicted trait values under the global and both local approaches. Across all analyses, the trait best predicted was pH, with a correlation between the true and the predicted value under global PLSR of 0.77. The trait with the weakest correlation across all analyses was CMS, with a correlation between the true and the predicted value under global PLSR of 0.32. 

Table 3 reports the RMSEV for the LOCAL approach for each combination of number of neighbours and number of PLSR factors. For pH, the lowest RMSEV was obtained using 100 neighbours and 10 PLSR factors, while for all the other traits the lowest RMSEV was obtained using 100 neighbours and 5 PLSR factors.

Comparing the performance of the global and the local approaches across the full dataset using RMSEV, with the exception of k20, the global approach outperformed the local approaches irrespective of the type of approach (LOCAL or local changepoint), or the settings of the method. Comparing the performance of the LOCAL and the local changepoint approaches, the local changepoint approach outperformed the LOCAL approach on the prediction of four traits: RCT, k20, a30, and CMS. 

**Figure 1:**
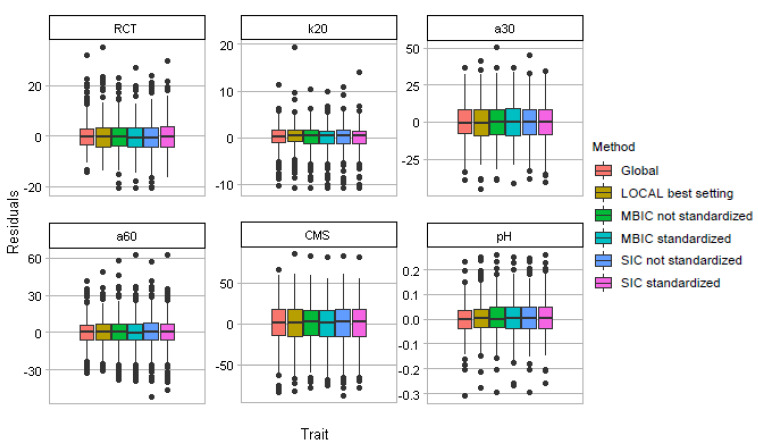
Boxplots of the residuals for each trait from global, LOCAL, and local changepoint approaches according to the alternative penalties tested on spectral data either standardised or unstandardised prior to neighbour selection.

Examining the residuals for each of the global and the local approaches (Figure 1) provides a deeper insight. The median of the residuals was consistent and close to zero across methods; however, the interquartile range of the residuals was generally smaller for the global approach.

### 3.2. Prediction for Spectra at the Edge of the Principal Component Space

**Figure 2:**
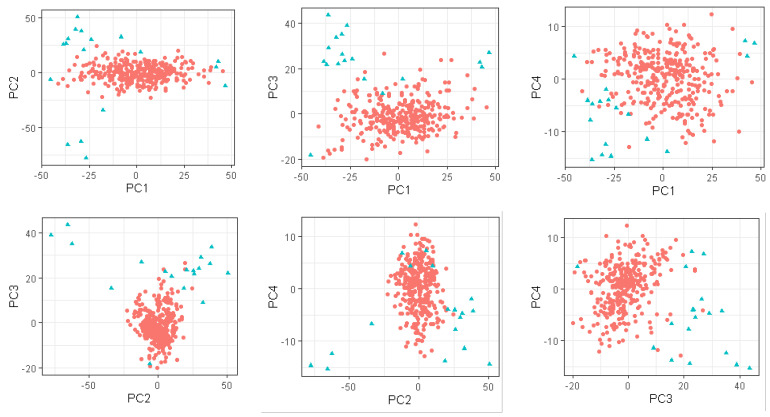
Spectra in the centre (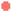) and on the edge (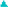) (the 5% greatest Mahalanobis distance from the centre, of the four-dimensional principal component space of standardised spectra.

Table 4 presents the prediction performance of the 5% of observations with the greatest Mahalanobis distance from the centre of the global PC space (Figure 2) using the global and local PLSR approaches. For these outlying observations, the RMSEV using the local changepoint approach was lower than the global RMSEV not only for k20, but also for CMS, where the RMSEV of the local changepoint approach was lower than the global RMSEV in the MBIC with the standardised spectra setting, and in the SIC with the non-standardised spectra setting. In the a30 analyses, r was greater for the local changepoint approach than the global approach using the MBIC with standardised spectra and SIC with non-standardised spectra settings; however, a lower RMSEV was achieved using the global approach. Using the LOCAL approach, the RMSEV was always higher than the RMSEV of the global approach. If the LOCAL and local changepoint approaches are compared for these 5% outlying observations, the local changepoint approach outperformed the LOCAL approach for five traits. When the prediction performance of the 10% of observations with the greatest Mahalanobis distance from the centre of the PC space were considered, prediction for only k20 was improved using the local changepoint approach compared to the global approach. 

### 3.3. Selection of the Number of Neighbours and Factors

**Figure 3:**
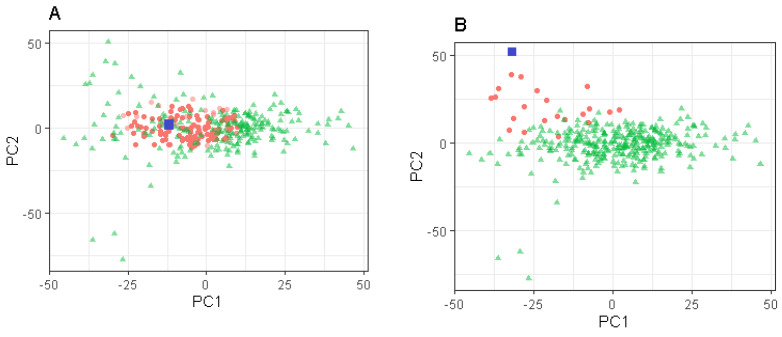
Neighbours selected according to the MBIC penalty after standardising the spectra for a target spectrum (blue square) that lies in the centre ((**A**) *n* = 133, red dots) and at the edge ((**B**) *n* = 21, red dots) of the four−dimensional principal component space. The green triangles represent the other spectra not selected as neighbours.

The number of neighbours selected by the changepoint analysis varied according to the method used for neighbour identification (type of penalty and if the spectra were standardised or not before the calculation of the PCs). The MBIC penalty generally resulted in a greater number of neighbours selected, with an average (range in parenthesis) selection of 58 (21–133) neighbours across the six traits studied when the spectra were standardised before the calculation of the PCs, compared to 60 (21–122) when the spectra were not standardised. Applying the SIC penalty, the average number of neighbours selected was 46 (21–92) when the spectra were standardised and 48 (21–100) when non-standardised. The greatest number of neighbours selected for a single observation was 133, when using the MBIC penalty with standardised spectra, and corresponded to the observation with the 25th smallest Mahalanobis distance from the centre of the PCs space (Figure 3A). The lowest number of neighbours selected for a single observation was 21 across all settings considered and corresponded to the observation with the 4th greatest Mahalanobis distance from the centre of the PCs space (Figure 3B).

Even when the same number of neighbours was selected for different predictands, different numbers of PLSR factors were identified. In Table 5, the mean, minimum, and maximum number of PLSR factors employed during the global PLSR analysis as well as the PLSR analyses for each local changepoint approach are presented. The results from the LOCAL approach are not presented, as a fixed number of PLSR factors (i.e., 5, 10, and 20) are used for all the observations. The number of factors selected by PLSR during the LOO CV analyses using the global approach slightly differed for each group of n−1 observations. In the local changepoint analyses, the number of PLSR factors selected as optimal for each group of neighbours varied and depended on the neighbours selected.

## 4. Discussion

Local approaches, whereby the points used to predict a target spectrum are specially selected based on their similarity to the predictand, have the potential to improve the prediction performance over global PLSR prediction in heterogeneous datasets and when non-linear associations are present between the spectra and the trait of interest [4,11]. While local approaches have improved prediction accuracy in many studies [13,14,15], the local approaches did not consistently benefit prediction accuracy in the homogeneous milk dataset analysed here. 

### 4.1. Selection of Spectra to Improve Prediction Accuracy 

**Figure 4:**
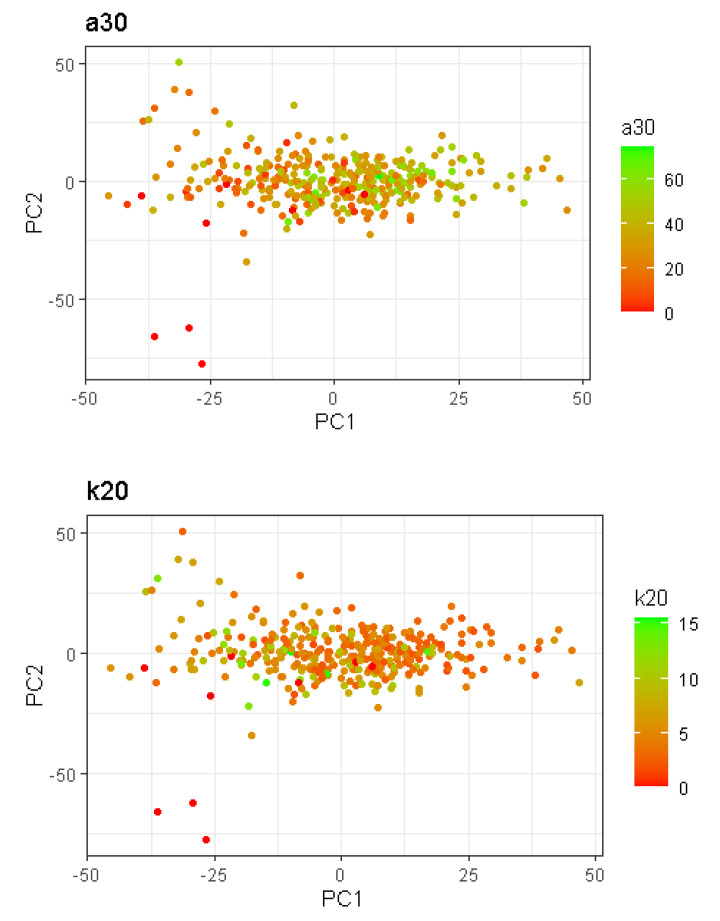
First and second principal components of the dataset, with each point representing a spectrum, and the points coloured according to the values of a30 and k20.

In this study, we applied an objective, data-driven approach to select the optimal spectra for prediction according to the characteristics of the predictand. This differs to previous studies which have applied the local approach, whereby the number of neighbours used during the PLSR analyses is constant for all the observations in a dataset, regardless of the differences between observations [4,29]. In fact, some predictands can have more similar observations in the data set used for prediction (thus more neighbours) than others. For example, considering the milk dataset used in the present study, the spectra with the most and least neighbours were compared (see Figure 3). The spectrum with the lowest number (21) of similar spectra as determined by the local changepoint approach was located in the edge of the PCs space (the fourth farthest point from the centre), and the spectrum with the greatest number of similar spectra (133) was located in the centre of the distribution (the 25th most central point). These neighbours were identified using the MBIC penalty after standardising the spectra. The objective of the present study was to take advantage of the characteristics of extreme spectra in the PC space using only similar spectra (the neighbours) for their prediction. However, for the milk dataset, no generalisable improvement was observed when selected samples were used for prediction over the global approach, whereby all the observations were used to determinate the prediction accuracy. One reason for this may be the homogeneous structure of the analysed milk data in the PC space, whereby there is no clear relationship or grouping structure between the phenotype values and observations in the PC space. Two examples are presented in Figure 4, where the phenotype values for a30 and k20 are coloured according to their phenotype value and plotted on the first two PCs; both traits demonstrate a homogeneous structure in the PC space, and there is no clear relationship between the spectra (in this case the first two PCs) and the phenotype. However, the local changepoint PLSR should be beneficial for a user with heterogeneous data, such as those considered in Lesnoff et al. [4], where distinct clusters are present in the PC space. 

When only the prediction performance of extreme observations (those with the 5% greatest Mahalanobis distances from the centre of the PC space) were analysed, the local changepoint approach improved the prediction performance compared to the global approach for two traits. Differently to the local changepoint approach, the LOCAL approach continued to have a greater RMSEV compared to the global approach for all the traits, and the local changepoint approach had a lower RMSEV than the LOCAL for five traits. These results highlight how the selection of different numbers of neighbours for each observation can be useful, in particular for those extreme observations which benefit from the selection of a lower number of, but more similar, neighbours.

Normally, when local approaches are employed, preliminary PLSR analyses are run to identify the best number of factors to use in successive analyses [4]. However, the best number of PLSR factors changes considerably across sets of neighbours, even if these are of the same size (Table 4). Furthermore, if the number of neighbours changes considerably according to the characteristics of the predictand, the best number of factors has to be identified for each group of neighbours. It can be seen in Table 4 how the median number of factors employed during the PLSR analyses is generally lower for the local changepoint approach compared to global approach, possibly related to the utilisation of less observations during the local PLSR analyses. Again, in the local changepoint analyses, the number of factors selected as optimal to run the PLSR model varies depending on the number and characteristics of the neighbours. A disadvantage of the changepoint approach is the potential increase in computation time; however, precautions can be employed, such as using k-fold CV instead of LOO CV for the identification of the number of factors. Leave-one-out CV was employed as a validation method here instead of external validation, in order to maximise the number of samples available as potential neighbours. Limitations of LOO CV include potential overfitting, thus producing biased results. However, as the objective here was to fairly compare different approaches, LOO CV was used across all methods. 

Whether or not the spectra were standardised prior to the calculation of the PCs led to differences in the neighbours selected by the changepoint approach. In the present study, after standardising the spectra, the first PC represented 52% of the variability of the spectra, while when the spectra were not standardised, the first PC identified 98% of the variability of the spectra. Consequently, when the neighbours are identified, and the spectra are not standardised, the first PC has an important effect on the process, while after standardising the spectra, the first PC still has a considerable impact, but not as strong as without standardising. The standardisation procedure can have an impact on any heterogeneity in the data, sometimes masking the presence of clusters, but on the contrary, if other PCs (after the first) are important for the identification of structure in the dataset, and the spectra are not standardised, these PCs have limited influence during the neighbours’ identification. 

It is thus necessary to consider all these different settings when running a local changepoint analysis, starting from the decision of whether to standardise the spectra and then the choice of the penalty to employ. 

### 4.2. Limit of Local Approaches in Homogeneous Datasets

The local approaches are typically applied to heterogeneous datasets, in which spectra from different materials are included in the same dataset [4,13]. For example, in Berzaghi et al. [13], four datasets were analysed using both global and LOCAL analyses. The LOCAL approach employed was that of Shenk and Westerhaus. The datasets of interest comprised a forage dataset (including hay, corn silage, small grain silage, and total mixed ration), a grain dataset (including barley, corn, oats, and wheat), a meat dataset (including meat and bone meal, fish meal, and poultry meal), and a feed dataset (including bakery products, mixed feed, poultry feed, and soya products). For all the datasets tested and across different traits, Berzaghi et al. [13] reported that the LOCAL approach outperformed the global analyses (i.e., in the analyses of the forage dataset the standard error of prediction was 12.70%, 17.53%, and 11.37% lower using the LOCAL compared to the global analysis for protein, acid detergent fibre (ADF), and dry matter, respectively). The heterogeneity of the spectra in the study of Lesnoff et al. [4] is evident in the plot of spectra in their first two PC spaces [4]; clusters of observations are easily identified. Conversely, in the dataset considered in the present study, composed only of milk spectra, no clusters of observations were evident in the PC space (Figure 2 and Figure 4). Although milk exhibits natural variability in its components, this variability did not carry through as heterogeneity in the spectral dataset. This was also confirmed by the poor predictions performed using the LOCAL approach in the present study.

It is thus important to know the dataset being analysed and to perform preliminary analyses to identify the characteristics of the dataset (heterogeneous or homogenous samples in the dataset, if clusters are present in the data) and consequently define the most suitable approach. For the specific milk dataset considered in this study, the application of the local approaches did not improve the accuracy in the majority of predictions; however, the prediction of particular traits did benefit from its application. As evidenced here, and to support the importance of always testing different approaches for trait prediction, Rabenarivo et al. [30] and Sila et al. [31] reported an improvement in the prediction performance using the local approach over the global approach for some traits, but not for others. 

## 5. Conclusions

The proposed local changepoint approach has the potential to improve prediction accuracy by objectively choosing the set of neighbours to use when developing the prediction equation by adapting the selection of the number of neighbours according to the characteristics of the predictand. Both the LOCAL and the local changepoint approach performed poorly for most traits within the milk dataset used in the present study, given its homogeneous nature. While the local changepoint approach showed improved prediction performance over the global approach for a minority of traits, the approach may have the potential to improve prediction performance in other datasets which exhibit heterogeneity. When considering extreme observations in the dataset (i.e., using the 5% of spectra with the greatest Mahalanobis distance from the centre of the global PC space), the performance of the local changepoint approach was slightly better than that of the global approach, and further outperformed the LOCAL approach, showing the potential improvement in prediction when fewer neighbours are selected for extreme observations.

## Figures and Tables

**Table 1:** Mean, standard deviation (SD), median, minimum (Min), and maximum (Max) values for the 6 technological traits studied.

Trait	Mean	SD	Median	Min	Max
RCT, min	17.24	6.61	17.75	0.00	29.75
k20, min	4.95	2.88	4.25	0.00	13.56
a30, mm	31.78	15.49	31.71	0.00	74.12
a60, mm	32.22	12.50	30.66	0.00	66.00
Casein micelle size, mm	170.20	24.89	166.00	109.10	244.00
pH	6.66	0.09	6.65	6.42	6.93

**Table 2:** Root mean square error of the validation samples (RMSEV) and correlation (r) between the true and predicted trait values using PLSR, using the global approach, the LOCAL approach, and using the local changepoint approach ^1^.

	Global		LOCAL		Local Changepoint							
Trait					MBIC ^2^				SIC ^2^			
					Spectra Standardised		Spectra Not Standardised		Spectra Standardised		Spectra Not Standardised	
	RMSEV	r	RMSEV	r	RMSEV	r	RMSEV	r	RMSEV	r	RMSEV	r
RCT, min	**5.74**	**0.51**	6.29	0.35	6.28	0.45	6.23	0.44	6.30	0.42	6.53	0.40
k20, min	2.55	0.47	2.71	0.42	2.60	0.47	**2.54**	**0.50**	2.63	0.46	2.59	0.48
a30, mm	**12.04**	**0.64**	13.25	0.54	13.01	0.56	13.59	0.52	13.27	0.54	13.23	0.55
a60, mm	**11.12**	**0.46**	11.41	0.38	12.46	0.32	12.38	0.33	12.69	0.30	12.69	0.30
CMS, mm	**24.17**	**0.32**	25.22	0.21	25.97	0.16	25.71	0.17	25.16	0.22	26.73	0.12
pH	**0.060**	**0.77**	0.065	0.73	0.070	0.70	0.068	0.72	0.070	0.70	0.070	0.70

^1^ Bold numbers identify the lowest RMSEV and highest correlation value for each trait. ^2^ MBIC = modified Bayesian information criterion; SIC = Schwarz information criterion.

**Table 3:** Root mean square error of validation sample (RMSEV) and correlation (r) between the true and predicted trait values across different LOCAL settings ^1^.

Traits	25 neigh 5 fact ^2^		25 neigh 10 fact		25 neigh 20 fact	
	RMSEV	r	RMSEV	r	RMSEV	r
RCT, min	7.09	0.32	8.49	0.29	10.11	0.20
k20, min	3.42	0.27	3.87	0.26	4.78	0.19
a30, mm	15.26	0.46	19.25	0.30	22.56	0.25
a60, mm	13.55	0.30	15.84	0.22	18.17	0.15
CMS, mm	30.08	0.19	34.99	0.17	41.11	0.15
pH	0.083	0.54	0.094	0.54	0.110	0.50
Traits	50 neigh 5 fact		50 neigh 10 fact		50 neigh 20 fact	
	RMSEV	r	RMSEV	r	RMSEV	r
RCT, min	6.56	0.34	6.88	0.39	8.97	0.31
k20, min	2.86	0.39	3.10	0.37	4.28	0.25
a30, mm	13.93	0.52	14.73	0.49	17.50	0.43
a60, mm	12.00	0.38	13.11	0.38	15.85	0.31
CMS, mm	26.63	0.21	28.68	**0.28**	34.42	0.23
pH	0.074	0.63	0.070	0.70	0.083	0.65
Traits	100 neigh 5 fact		100 neigh 10 fact		100 neigh 20 fact	
	RMSEV	r	RMSEV	r	RMSEV	r
RCT, min	**6.29**	0.35	6.34	**0.41**	7.54	0.37
k20, min	**2.71**	**0.42**	2.81	0.41	3.49	0.34
a30, mm	**13.25**	0.54	13.55	**0.55**	16.21	0.44
a60, mm	**11.41**	0.43	11.53	**0.47**	13.09	0.44
CMS, mm	**25.22**	0.21	26.55	0.26	31.36	0.24
pH	0.092	0.65	**0.065**	**0.73**	0.074	0.69

^1^ Bold numbers identify the lowest RMSEV and highest correlation value for each trait. ^2^ neigh = number neighbours; fact = number of PLSR factors.

**Table 4:** Root mean square error of validation samples (RMSEV) and correlation (r) between the true and predicted trait values for the observations with the 5% greatest Mahalanobis distance from the centre of the principal component space using global, LOCAL, and local changepoint approaches ^1^.

	Global		LOCAL		Local Changepoint							
Trait					MBIC ^2^						SIC ^2^	
					Spectra Standardised		Spectra Not Standardised		Spectra Standardised		Spectra Not Standardised	
	RMSEV	r	RMSEV	r	RMSEV	r	RMSEV	r	RMSEV	r	RMSEV	r
RCT, min	**4.76**	**0.82**	9.39	0.27	8.23	0.62	8.23	0.62	6.87	0.64	9.43	0.48
k20, min	2.88	0.52	5.03	0.00	2.17	0.75	2.14	0.77	2.18	0.76	**2.00**	**0.79**
a30, mm	**8.73**	0.82	16.11	0.66	9.41	**0.85**	12.81	0.74	11.70	0.75	9.55	0.84
a60, mm	**7.46**	**0.87**	8.92	0.82	8.80	0.84	7.51	0.87	8.52	0.85	8.06	0.85
CMS, mm	23.75	0.49	27.06	0.29	23.47	0.53	24.50	0.48	24.84	0.43	**22.90**	**0.54**
pH	**0.072**	**0.66**	0.076	0.66	0.091	0.59	0.091	0.60	0.082	0.65	0.094	0.55

^1^ Bold numbers identify the lowest RMSEV and highest correlation value for each trait. ^2^ MBIC = modified Bayesian information criterion; SIC = Schwarz information criterion.

**Table 5:** Median (range in parentheses) number of factors used to run the global PLSR, as well as the median (range in parentheses) number of factors used to run local changepoint PLSR. Results from the LOCAL approach are not reported, as a fixed number of PLSR factors are used for all the observations.

	Global	Local			
Trait		MBIC		SIC	
		Spectra Standardised	Spectra Not Standardised	Spectra Standardised	Spectra Not Standardised
RCT, min	13 (13–14)	5 (1–20)	5 (1–20)	4 (1–20)	4 (1–20)
k20, min	3 (3–13)	3 (1–19)	3 (1–18)	2 (1–20)	2 (1–20)
a30, mm	13 (10–14)	4 (1–20)	3 (1–20)	3 (1–20)	3 (1–20)
a60, mm	9 (8–9)	3 (1–20)	4 (1–20)	3 (1–20)	3 (1–20)
CMS, mm	11 (9–13)	2 (1–20)	2 (1–20)	2 (1–20)	2 (1–20)
pH	14 (14–14)	9 (1–20)	9 (1–20)	8 (1–20)	8 (1–20)
